# Metabolomic analysis of swainsonine poisoning in renal tubular epithelial cells

**DOI:** 10.3389/fvets.2024.1387853

**Published:** 2024-05-21

**Authors:** Shuhang Zhang, Yingqingqing Zhang, Hai Yin, Yiling Liu, Lihui Tang, Yanli Zhu, Pinzhi Sun, Kexin Wu, Baoyu Zhao, Hao Lu

**Affiliations:** College of Veterinary Medicine, Northwest A&F University, Yangling, Shaanxi, China

**Keywords:** swainsonine, metabonomics, rat renal tubular epithelial cells, bile secretion, cytochrome P450

## Abstract

Locoweed is a poisonous plant widely present in grasslands around the world. Swainsonine (SW), an indole alkaloid that, is the main toxic component of the locoweed. To understand the mechanism of SW-induced toxicity and to delineate the metabolic profile of locoweed poisoning we performed the LC–MS/MS untargeted metabolomic study to analyze metabolites in SW-treated renal tubular epithelial cells (0.8 mg/mL, 12 h) and in order to identify the SW-induced metabolomic changes. The analysis identified 2,563 metabolites in positive ion mode and 1,990 metabolites in negative ion mode. Our results showed that the metabolites were mainly benzenoids, lipids and lipid-like molecules, nucleosides, nucleotides, and analogs, organic acids, and derivatives. The differential metabolites were primarily enriched in pathways involving bile secretion, primary bile acid biosynthesis, riboflavin metabolism, ferroptosis, drug metabolism-cytochrome P450, and primidine metabolism. We have screened out substances such as swainsonine, 3alpha,7alpha-Dihydroxy-5beta-cholestanate, 2-Hydroxyiminostilbene, and glycochenodeoxycholate, which may have the potential to serve as biomarkers for swainsonine poisoning. This study provides insights into the types of metabolomic alteration in renal tubular epithelial cells induced by swainsonine.

## 1 Introduction

Locoweed, a plant species with a global distribution (1), has been documented in various countries including the United States (2), Brazil (3), Argentina (4), Australia (5), and China (6). The earliest instances of poisonous plant poisoning diseases in the western region of the country were documented in the United States in 1873 (7). In China, the distribution of locoweed is mainly concentrated in Qinghai, Xinjiang, Tibet, Gansu, and other regions (8). Swainsonine (SW) is an indolizidine alkaloid, which is the main toxic component of locoweed (9–11). SW-induced toxicity is primarily characterized by the inhibition of α-mannosidase activity and the induction of widespread cellular vacuolar degeneration (12, 13). Previous studies have unequivocally demonstrated that SW can inflict damage on multiple organs, encompassing the cerebrum, cerebellum, liver, kidneys, pancreas, and thyroid gland (1). Notably, renal injury is observed at relatively lower dosages or at earlier time points (4, 14).

Metabolites are important characteristics of an organism’s phenotype, understanding the metabolomics provides important insights for the biological processes and their underlying mechanisms. Metabolomics is the systematic quantification and analysis of all small molecules (15) present in biological samples such as cells, tissues, or biological fluids. It involves the qualitative and quantitative characterization of these substances and their comprehensive analysis. Metabolomics has the ability to directly capture the molecular phenotype of a species (16) as well as the functional state of an individual (17–19). Through this analysis, metabolites of biological significance and statistically significant differences can be identified thus facilitating disease diagnosis and metabolite analysis. Metabolomics technology has been extensively employed in the identification of biomarkers for disease diagnosis and screening. Studies have utilized this technology to screen for specific metabolite associated with depression (20), neurodegenerative diseases (21), venous thromboembolism (22), osteoporosis (23), and diabetes mellitus (24). Currently, the metabolomics research surrounding locoweed and SW remains scant, with the bulk of studies focusing on elucidating the intricate relationship between the secondary metabolites of endophytic fungi and the biosynthesis of SW. Unfortunately, research papers exploring the intoxication mechanisms of SW are highly limited, with only a solitary study identified. In this study, the author cleverly harnessed targeted metabolomics and high-throughput sequencing techniques to reveal that SW has the capacity to profoundly alter bile acid metabolism and disrupt the delicate balance of intestinal microbiota in mice, ultimately triggering inflammatory reactions in the liver (25). Nevertheless, our understanding of the kidney toxicity and associated metabolites triggered by SW remains tenuous, necessitating further investigation in this vital area.

Currently, the prevention and control of livestock poisoning primarily rely on the implementation of preventive measures, as there is currently no specific antidote drug available for affected animals. As the early stage of locoweed poisoning is reversible, timely intervention can help mitigate the symptoms of locoweed poisoning by feeding non-toxic pasture grasses. Therefore, timely identification of the metabolic changes due to the locoweed poisoning is important for effective intervention. At present, studies on SW primarily focuses on its biosynthesis and toxicity mechanisms, with limited reports on the metabolic pathways it participates in after entering cells. Previous research has indicated that locoweed poisoning primarily affects the kidney, causing severe damage to this organ and the renal epithelial cells, especially the proximal tubular epithelial cells, are the most significantly affected (4, 14). In this study, rat primary renal tubular epithelial cells were utilized as an experimental model to investigate the metabolomic perturbation following locoweed poisoning. Untargeted metabolomics technology was employed to analyze the metabolic profile of SW-induced alteration in renal tubular cells in order to aid the diagnosis of locoweed poisoning. The identification of these metabolomic changes is important for the early detection and prevention of locoweed poisoning in livestock.

## 2 Materials and methods

### 2.1 Ethical approval

All procedures were conducted in accordance with the Code of Ethics and approved by the Laboratory Animal Management and Ethics Committee of Northwest A&F University, Project number: XN2023-1007. All efforts were made to minimize the suffering of animals.

### 2.2 Chemical reagents and instrumentation

Swainsonine (provided by the Laboratory of Animal Toxicology Team, Northwest A&F University, purity >98%), methanol (Merck, Germany), acetonitron (Merck, Germany), L-2-chlorophenylalanine (Aladin, China), formic acid (TCI, Japan), ultra-high performance liquid phase (Waters, United States), High resolution mass spectrometry (Waters, United States), chromatographic column (Waters, Unites States), and Cell incubator (Thermo, United States).

### 2.3 Cell culture and treatment

Primary rat renal tubular epithelial cells were obtained by tissue block culture, the detailed operational steps are as follows.

Euthanize the rats by decapitation, and aseptically extract the kidneys. Delicately remove the capsule and separate the cortical portion. Cut the cortical tissue into 1 mm^3^ tissue blocks. Thoroughly rinse the tissue blocks with PBS buffer until the supernatant becomes clear and free of turbidity. Centrifuge the tissue blocks at 1,000 rpm for 3 min. Add 0.1% type IV collagenase to the tissue blocks and incubate the mixture at 37°C for 1 h to disperse the tissue. To terminate the enzymatic digestion, add an equal volume of culture medium to the mixture. Pass the content of the centrifuge tube through a 100-mesh screen to collect the filtered solution. Repeat the filtration process using a 400-mesh screen and collect the upper layer material, which represents the renal tubule segments. Resuspend the renal tubule segments in an appropriate amount of culture medium. Transfer the resuspended renal tubule segments to culture dishes and incubate them in a cell culture incubator. Replace the culture medium every 48 h to maintain the optimal growth conditions.

When the cells were fused to 80%, SW is dissolved in the culture medium to achieve a concentration of 0.8 mg/mL. Subsequently, the SW-containing culture medium is filtered through a 0.22 μm filter membrane to ensure sterility. The sterilized medium is then applied to the cells and incubated for 12 h.

The cell culture medium used in our study was Dulbecco’s Modified Eagle Medium (Gibco, America, 12800-017), supplemented with 15% Fetal Bovine Serum Standard (Newzerum, New Zealand, FBS-S500) for cultivation, without the addition of any other substances.

### 2.4 Metabolites extraction

The extraction solution with internal label (Vmethanol:Vacetonitrile = 1:1, internal standard concentration 20 mg/L) was prepared, and the extraction solution was added three times (300, 300, and 400 μL), and the cell samples were completely transferred into the EP tube, and vortex mixed for 30 s. Add steel beads, process for 10 min on a 45 Hz grinder, sonicate for 10 min (ice-water bath); leave at −20°C for 1 h; centrifuge at 12,000 rpm for 15 min at 4°C, and extract the supernatant (500 μL); dry the extract in a vacuum concentrator; after drying, add 160 μL of the extract solution (V water:V acetonitrile = 1:1) to reconstitute the extract solution; vortex for 30 s, and sonicate for 10 min in an ice-water bath. The extract was centrifuged at 12,000 rpm for 15 min at 4°C. 120 μL of the supernatant was taken into the injection bottle, and 10 μL of each sample was mixed to form quality control samples (QC) for the assay.

### 2.5 LC–MS/MS analysis

The liquid-mass spectrometry system for metabolomics analysis consisted of an Acquity I-Class PLUS (Waters, United States) ultra-high performance liquid chromatography (UHPLC) tandem with a Waters Xevo G2-XS QTOF (Waters, United States) high-resolution mass spectrometer. The column used was Acquity UPLC HSS T3 column (1.8 μm, 2.1 mm × 100 mm) (Waters, United States). The mobile phase of positive ion mode (POS) and negative ion mode (26) were the same: mobile phase A: 0.1% formic acid aqueous solution; mobile compositions B: 0.1% formic acid acetonitrile, and the injection volume was 1 μL. The elution gradients were: 98% mobile phase A, 2% mobile phase B, 0–0.25 min; 2% mobile phase A, 98% mobile phase B, 10–13 min; 98% mobile phase A, 2% mobile phase B, 13.1–15 min; and the flow rate was 400 μL/min. The high-resolution mass spectrometer (Xevo G2-XS QTOF, Waters, United States) is capable of primary and secondary mass spectrometry data acquisition in MSe mode under the control of acquisition software (MassLynx V4.2, Waters, United States). Dual-channel data acquisition for both low and high crash energy is performed in each data acquisition cycle. The low collision energy was 2 V, the high collision energy interval was 10–40 V, and the scanning frequency was 0.2 s. The parameters of the ESI ion source were as follows: capillary voltage: 2,500 V (positive ion mode) or −2,000 V (negative ion mode); cone-well voltage: 30 V; temperature of the ion source: 100°C; temperature of the desolvent gas was 500°C; flow rate of the blowback gas was 50 L/h; flow rate of the desolvent gas was 800 L/h; and mass-to-nucleus ratio (m/z) acquisition range was 50–1,200. Flow rate: 800 L/h; mass-to-core ratio (m/z) acquisition range 50–1,200.

### 2.6 Characterization and quantification of metabolites

The raw data collected by MassLynx V4.2 were processed by Progenesis QI software for peak extraction and peak alignment, and then identified based on the Progenesis QI software online METLIN database, Human Metabolome Database (HMDB), and Biomark’s self-built library, and the theoretical fragments were also identified, and the deviation of mass number of parent ions is 100 ppm, and that of fragments is 50 ppm or less. The deviation of the parent ion mass number is 100 ppm and the deviation of the fragment ion mass number is 50 ppm or less (27).

### 2.7 Classification and functional annotation of metabolomes

The metabolites were annotated using the HMDB database^1^ to obtain superclass and class information; the pathways in which the identified metabolites were found were annotated using the Kyoto Encyclopedia of Genes and Genomes (KEGG) database^2^ to annotate the pathways in which the identified metabolites were found.

### 2.8 Data processing and analysis

Principal component analysis, which downscales the dimension of high-throughput metabolic data and categorizes them by the similarity of their principal components, is an unsupervised classification mode that responds to the overall distribution of samples among groups and the magnitude of differences between samples within groups. The supervised discriminant analysis statistical method of partial least squares regression orthogonal projections to latent structures-discriminant analysis (OPLS-DA) was used to visualize within-group differences by filtering out orthogonal variables unrelated to categorical variables. To check the reliability of the OPLS-DA model, a permutation test is required. The groupings of the samples were randomly disrupted and the OPLS-DA modeling was performed and the evaluation parameters coefficient of determination for Y (R^2^Y) and predictability for Y (Q^2^Y) were obtained according to the new groupings. Variable importance in projection (VIP) reflects both the loading weight of each metabolite in the model and the variability of the response explained by that metabolite, and can be used for ANOVA. In order to analyze the metabolic patterns of metabolites under different experimental conditions, all the metabolites with differences between the obtained comparison pairs were clustered into classes with the same or similar metabolic patterns for hierarchical clustering analysis.

## 3 Results

### 3.1 Quality control of survey data

**Figure 1:**
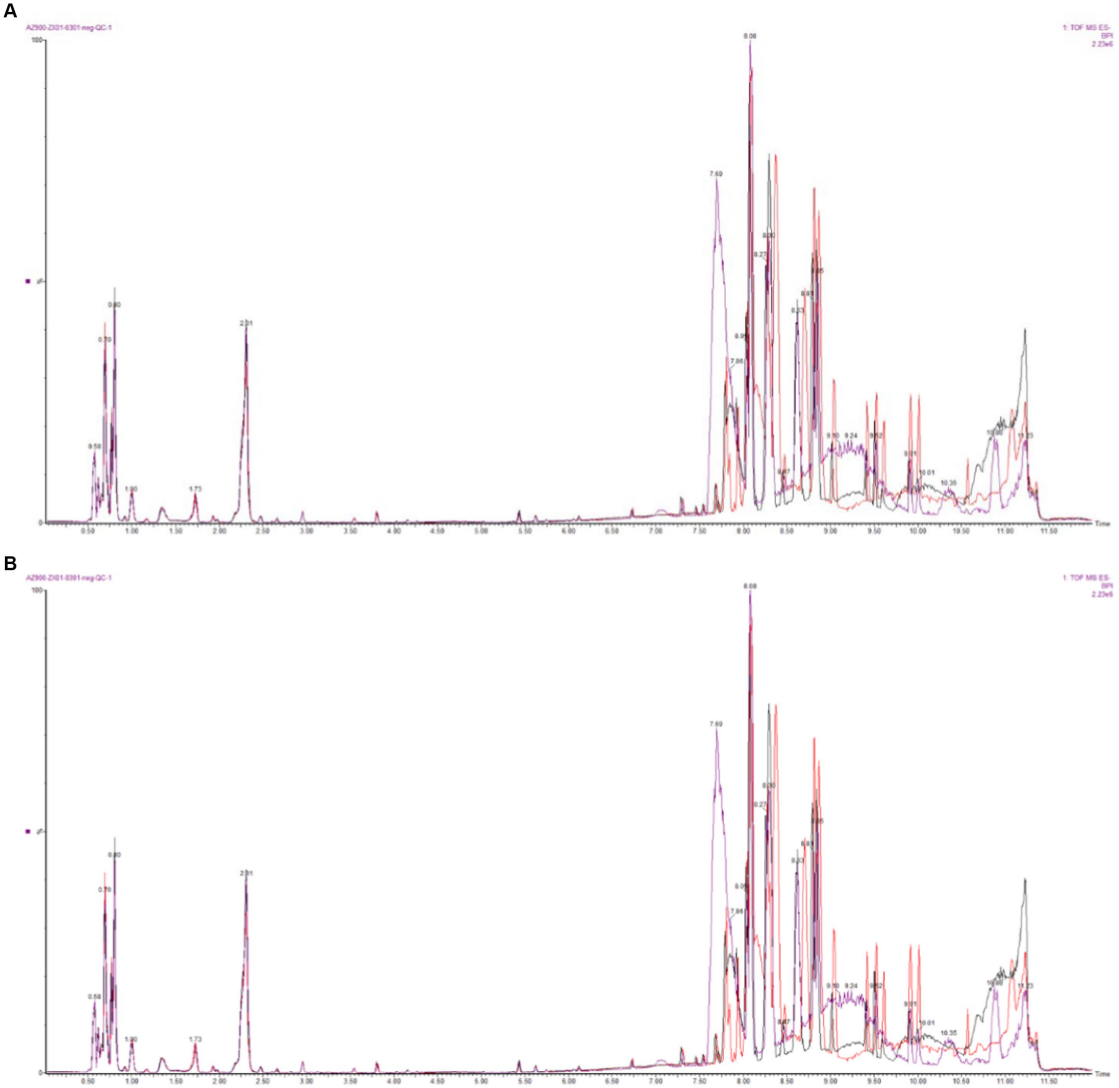
Base peak intensity (BPI) overlapping diagram of QC samples (**A**: positive ion mode; **B**: negative ion mode).

The base peak intensity (BPI) charts monitor the strongest peaks in each chromatogram. The strongest peak intensity at each point in the analysis is shown (Figure 1). The BPI from the QC sample has a good overlap of peak retention time and peak area, indicating good instrument stability. The positive ion mode 17,312 peaks were identified and 2,563 metabolites were annotated, of which 94.25% had relative standard deviation (RSD) ≥ 0.7; in the negative ion mode 7,825 peaks were identified and 1,990 metabolites were annotated, of which 86.81% had RSD ≥ 0.7 (Table 1). If the RSD is greater than 60%, it is an indication that the sample is reliable and can be used for follow-up testing.

**Table 1:** List of quality control indicators.

Mode	Number_of_peaks	Number_of_metabolites	QC_RSD_percent (≥0.7)
pos	17,312	2,563	94.25%
neg	7,825	1,990	86.81%

### 3.2 Principal component analysis

**Figure 2:**
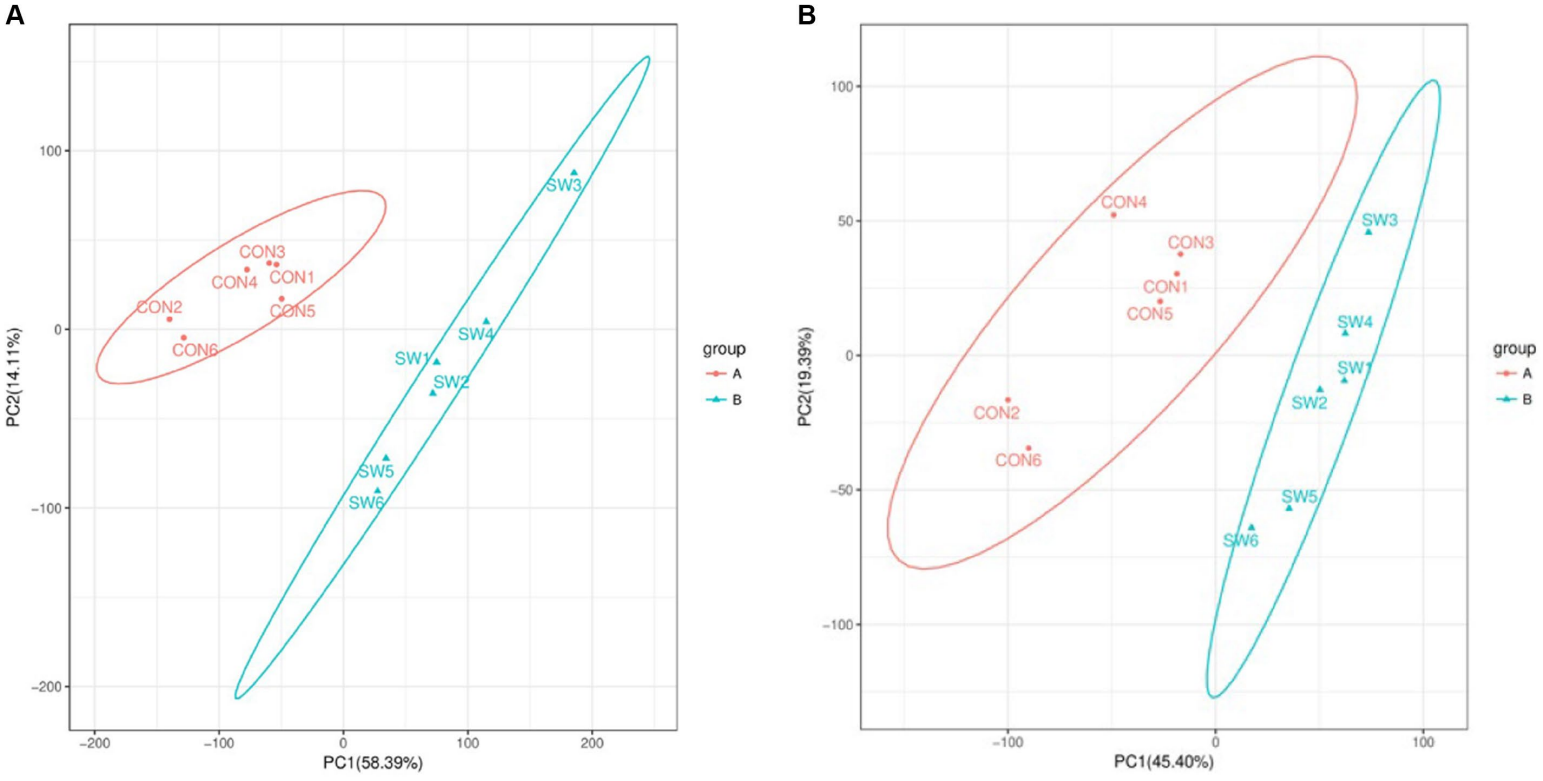
Principal component analysis (PCA) of all samples (**A**: positive ion mode; **B**: negative ion mode). Where the *x*-axis indicates the first principal component, the *y*-axis indicates the second principal component, and the percentage axis indicates the percentage contribution of that principal component to the sample variance. Each point on the graph represents a sample, samples in the same group are shown in the same color and samples in different subgroups are shown in different colors. Ellipses represent 95% confidence intervals.

By performing principal component analysis (PCA) on the samples, we can preliminarily understand the overall metabolic differences between the samples of each group and the degree of variation between the samples within the group. After PCA analysis of the total samples, the results (Figure 2) showed that the two groups of samples were divided into two different groups, and each sample was within the ellipse of the 95% confidence interval, indicating that the samples in the group had good repeatability. The two ellipses were independent of each other and there was no overlapping area, indicating that there was a large difference between the two groups of samples.

### 3.3 Orthogonal partial least squares discriminant analysis

**Figure 3:**
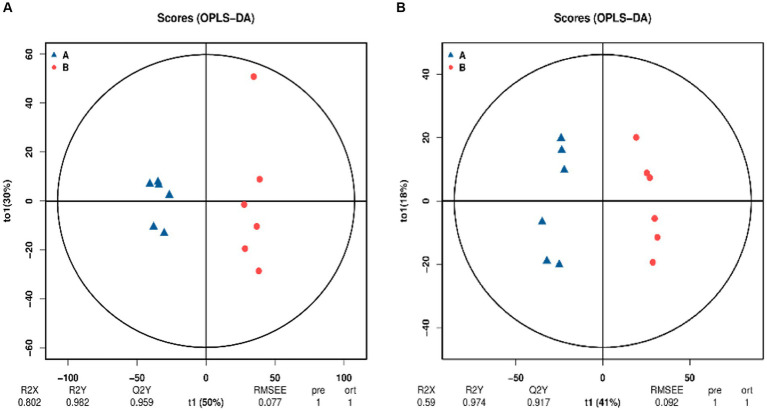
Orthogonal projections to latent structures–discriminant analysis (OPLS-DA) score chart (**A**: positive ion mode; **B**: negative ion mode). The blue point A in the image is the control group and the red point B is the SW treatment group. The *x*-axis (t1) represents the prediction component (the between-group variance component), the *y*-axis (t2) represents the orthogonal component (the within-group variance component), and the cross *y*-axis percentage represents the component’s proportion of the total variance. The model parameters are listed below, including R^2^X, R^2^Y, Q^2^Y, root mean square error (RMSEE), pre (number of predicted components), and ort (number of orthogonal components).

Orthogonal partial least squares discriminant analysis (OPLS-DA) is a supervised analysis that can better screen for differential metabolites by excluding influences that are not relevant to the study. OPLS-DA analysis was performed on the control and SW treatment groups and the results are shown in Figure 3. Under the two modes, the samples of the two groups were clearly grouped, indicating that the metabolites of the two groups were significantly different.

**Figure 4:**
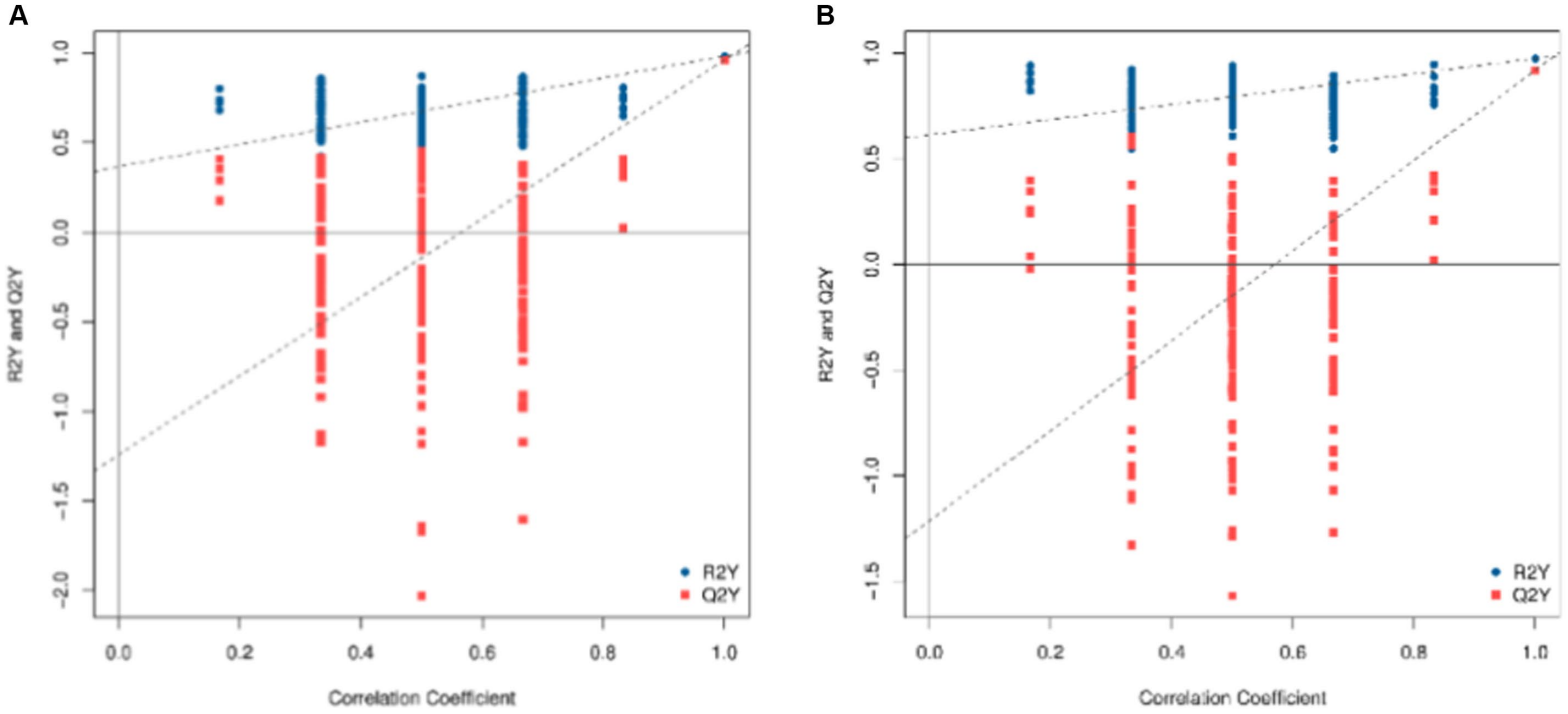
Replacement test diagram of OPLS-DA model (**A**: positive ion model; **B**: negative ion mode). In this image, the *X*-axis represents the correlation between the replacement group and the original model group, the *Y*-axis represents the value of R^2^Y or Q^2^Y (where Q^2^Y and R^2^Y of 1 are the values of the original model), the blue point and the red point represent the R^2^Y and Q^2^Y of the model after replacement, respectively, and the two dotted lines represent the regression line fitted by R^2^Y and Q^2^Y.

To check the reliability of the OPLS-DA model, a replacement test is required. This means that the grouping of the samples is randomly scrambled, and the OPLS-DA is modeled according to the scrambled grouping and its R^2^Y and Q^2^Y are calculated. After many iterations, the results of multiple modeling are plotted on a scatterplot, as shown in Figure 4, where Q^2^Y > 0.9 indicates that the model is excellent. The intercept of the Q^2^Y fitting regression line is negative, indicating that the model does not overfit; the slope of the Q^2^Y fitting regression line is positive, indicating that the model is meaningful, and the blue point is mostly above the red point, indicating that the independence of the modeling training set and test set is good. In the negative ion mode, the blue point overlaps the red point. This shows that the independence of the negative ion mode is not as good as that of the positive ion mode.

### 3.4 Metabolite classification and functional annotation

**Figure 5:**
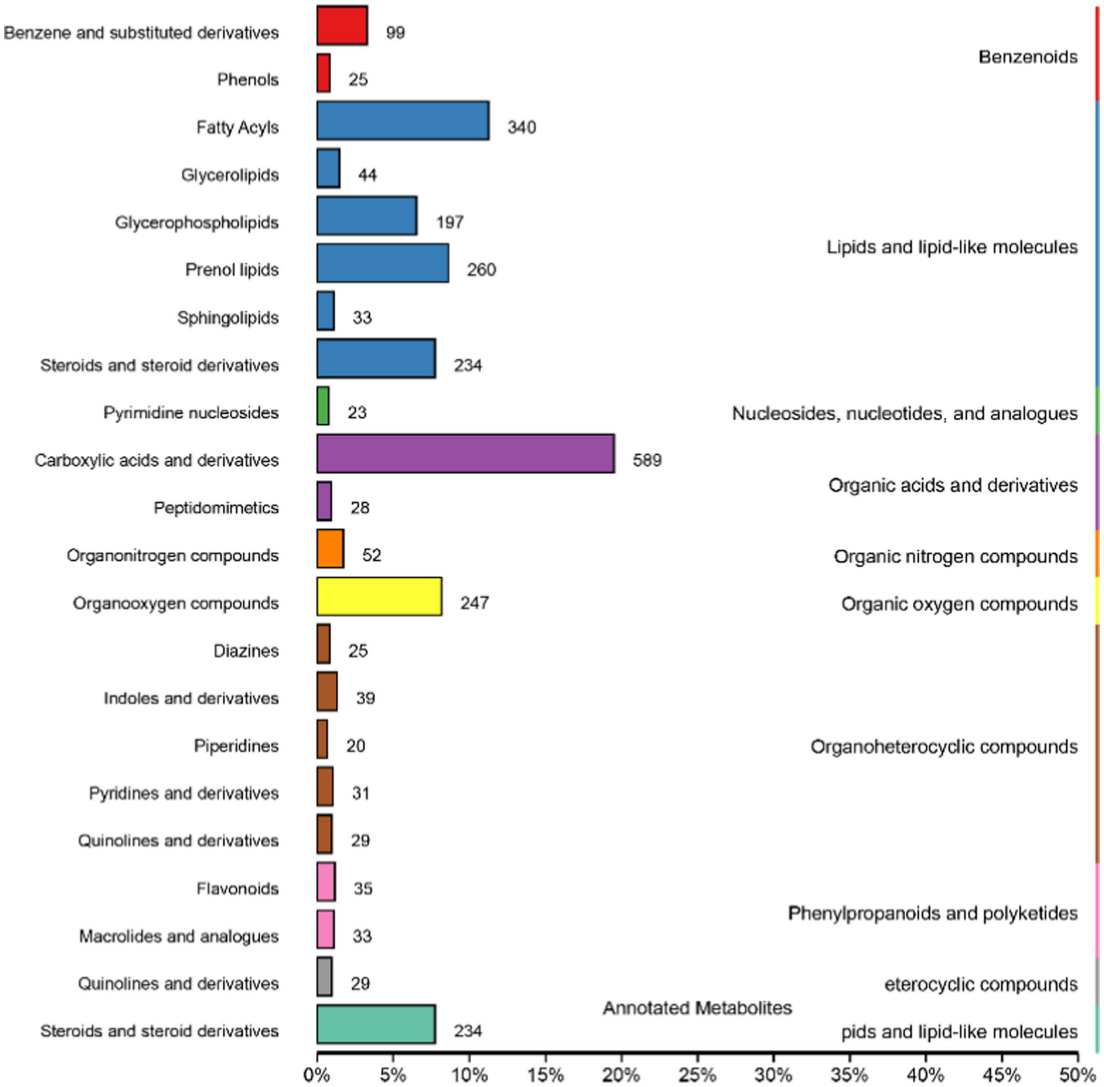
Human Metabolome Database (HMDB) classification summary. Items in the same box in the image represent HMDB level classification information, corresponding to the super class and class information of the HMDB database. Column length represents the number of metabolites annotated by the classification.

Metabolites in positive and negative ion mode are combined and annotated in HMDB as shown in Figure 5. The metabolites mainly include benzenoids, lipids and lipid-like molecules, nucleosides, nucleotides and analogs, organic acids and derivatives, organic nitrogen compounds, organic oxygen compounds, organoheterocyclic compounds, phenylpropanoids and polyketides, eterocyclic compounds, and pids and lipid-like molecules.

**Figure 6:**
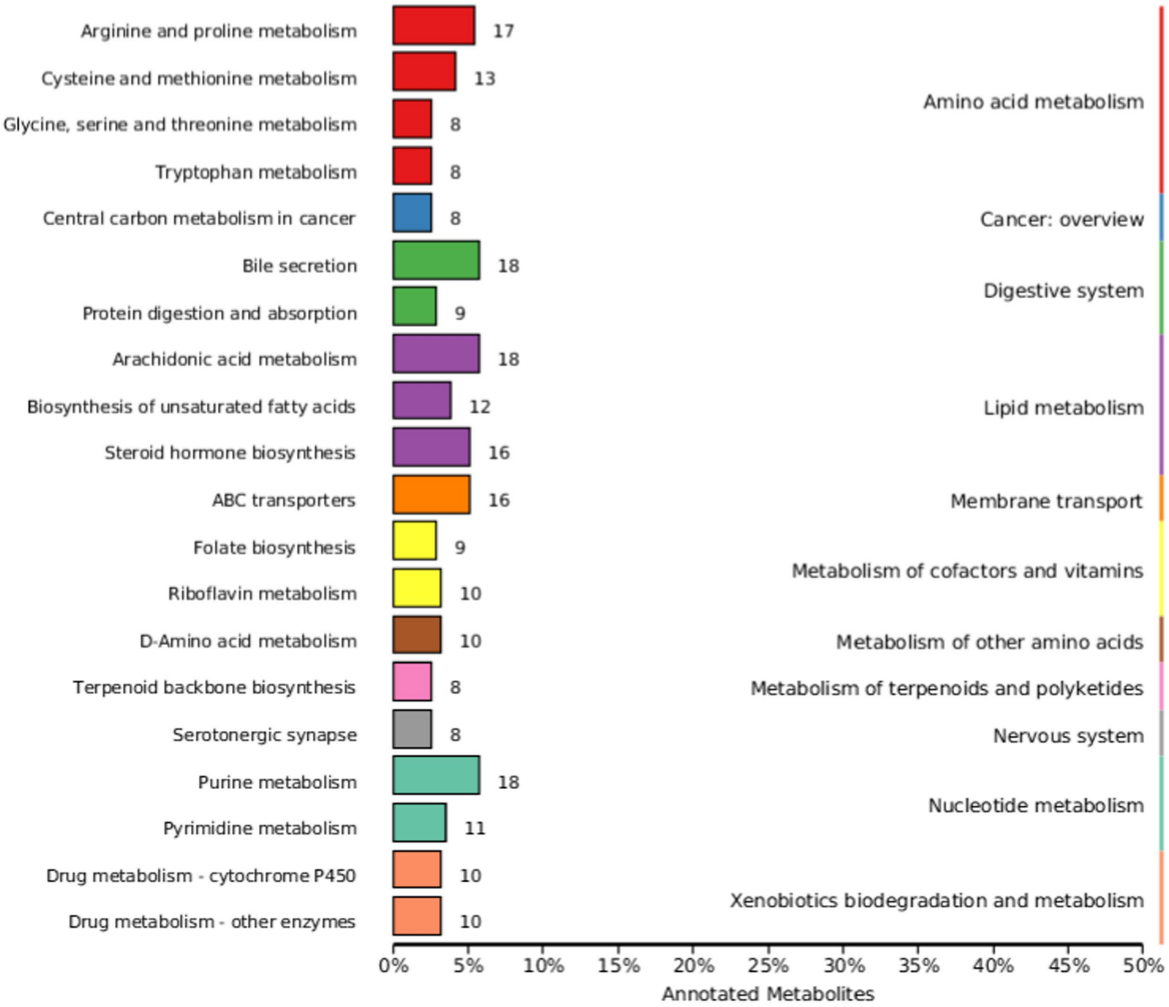
Kyoto Encyclopedia of Genes and Genomes (KEGG) database classification summary. The entries under the same box in the image represent the level classification notes of the KEGG pathway, corresponding to KO pathway Level 1 and KO pathway Level 2. The length of the column indicates the number of metabolites annotated by the pathway.

The KEGG database is a major public database related to metabolic pathways, which can be used for metabolic analysis *in vivo*. KEGG database is used to annotate all identified metabolites. Figure 6 shows the most annotated information (top 20 displayed) of Knock Out (KO) pathway level 2. The main metabolic pathways affected include amino acid metabolism, cancer: overview, digestive system, lipid metabolism, membrane transport, metabolism of cofactors and vitamins, overview, digestive system, lipid metabolism, membrane transport, metabolism of cofactors and vitamins, metabolism of other amino acids, metabolism of terpenoids and polyketides, nervous system, metabolism of other amino acids, metabolism of terpenoids and polyketides, nervous system, Nucleotide metabolism, and Xenobiotics biodegradation and metabolism.

### 3.5 Differential metabolite analysis

Based on the results of OPLS-DA, the VIP of the OPLS-DA model can be obtained by multivariate analysis, and the metabolites of different varieties or tissues can be initially screened out. The screening criteria are ① FC ≥ 1; ② VIP ≥ 1; and ③ *p* < 0.05.

By combining metabolites in positive and negative ion mode, a total of 2,170 differential metabolites were identified, of which 1,342 were upregulated and 828 were downregulated. Details of the differential metabolites are shown in Supplementary Table 1.

#### 3.5.1 Difference multiple analysis

The Fold Changes were compared after qualitative and quantitative analysis of the detected metabolites Table 2 shows the results of upregulated and downregulated log_2_FC of the top 20 metabolites in the test group compared with the control group after log conversion treatment for differential metabolite multiple. Among them, the top 5 metabolites with the highest upregative difference multiple were Ttricosanoylglycine, 1-Tricosanol, Sambacolignoside, Kanzonol T, and Nemonoxacin, respectively. The top 5 metabolites with the largest down-variance were 2-(Ethylamino)-4,5-dihydroxybenzamide, Cinobufotalin, Undecylprodigiosin, Hypochoeroside A, and Muzanzagenin.

**Table 2:** Results of differential metabolite multiple analysis.

ID	Name	Formula	m/z	Retention time (min)	log_2_FC	Regulated
neg_6622	Tricosanoylglycine	C_25_H_49_NO_3_	456.37	7.88	37.11	Up
pos_7083	1-Tricosanol	C_23_H_48_O	379.33	3.28	36.80	Up
neg_1289	Sambacolignoside	C_43_H_54_O_22_	921.29	0.70	36.29	Up
pos_1086	Kanzonol T	C_25_H_26_O_7_	915.31	0.70	35.38	Up
pos_12100	Nemonoxacin	C_20_H_25_N_3_O_4_	354.18	4.14	35.34	Up
pos_11933	2-Benzylidene-1-heptanol	C_14_H_20_O	222.19	4.08	35.22	Up
pos_11705	Lotaustralin	C_11_H_19_NO_6_	279.15	4.02	35.19	Up
pos_13943	FMNH2	C_17_H_23_N_4_O_9_P	481.11	5.43	35.07	Up
neg_1292	Probenecid Glucuronide	C_19_H_27_NO_10_S	981.29	0.70	35.06	Up
pos_14727	(Z)-7-Hexadecen-1,16-olide	C_16_H_28_O_2_	527.41	6.94	35.01	Up
neg_1212	(13Z,16Z)-Docosadienoyl-CoA	C_43_H_74_N_7_O_17_P_3_S	1120.39	0.70	34.86	Up
pos_14363	Herculin	C_16_H_29_NO	234.22	6.30	34.58	Up
pos_13944	Coriandrin	C_13_H_10_O_4_	483.11	5.43	34.37	Up
pos_14513	N2-Galacturonyl-L-lysine	C_12_H_22_N_2_O_8_	340.17	6.67	34.19	Up
pos_17309	Pyridinoline	C_18_H_28_N_4_O_8_	451.18	9.52	34.13	Up
pos_13603	3-Heptylpyridine	C_12_H_19_N	195.19	5.06	34.10	Up
pos_13914	4-Amino-3-hydroxybutanoylcarnitine	C_11_H_22_N_2_O_5_	280.18	5.37	34.03	Up
pos_13931	Pantoyllactone glucoside	C_12_H_20_O_8_	331.08	5.41	33.75	Up
pos_11369	2-Amino-5-formylamino-6-(5-phospho-D-ribosylamino)pyrimidin-4(3H)-one	C_10_H_16_N_5_O_9_P	399.11	3.93	33.68	Up
neg_1285	(1-(2-(Methylsulfonamido)ethyl)piperidin-4-yl)methyl 5-fluoro-2-methoxy-1H-indole-3-carboxylate	C_19_H_26_FN_3_O_5_S	899.31	0.70	33.60	Up
neg_4335	Linalool oxide D 3-(apiosyl-(1->6)-glucoside)	C_21_H_36_O_11_	463.22	3.27	−29.65	Down
neg_1374	Ile-Val-Val	C_16_H_31_N3O_4_	364.20	0.75	−29.76	Down
neg_3616	Rotundifoline	C_22_H_28_N_2_O5	421.17	2.63	−29.96	Down
neg_1905	Labetalol	C_19_H_24_N_2_O_3_	349.15	1.91	−30.15	Down
neg_4644	Bursin	C_14_H_25_N_7_O_3_	374.17	3.52	−30.19	Down
neg_1726	2-Isopropyl-5-methoxypyrazine	C_8_H_12_N_2_O	349.19	1.14	−30.21	Down
neg_5099	Periplocin	C_36_H_56_O_13_	741.37	3.97	−30.51	Down
neg_1744	Histidyltryptophan	C_17_H_19_N_5_O_3_	340.14	1.20	−30.55	Down
neg_4478	LysoPE (20:4(5Z,8Z,11Z,14Z)/0:0)	C_25_H_44_NO_7_P	536.25	3.38	−30.62	Down
neg_5581	CDP-DG [a-21:0/20:4(5Z,8Z,11Z,14Z)-OH (20)]	C_53_H_91_N_3_O_16_P_2_	1108.56	4.85	−30.65	Down
neg_1177	2′-C-Methylcytidine	C_10_H_15_N3O_5_	513.21	0.68	−31.05	Down
neg_4257	N-Docosahexaenoyl Aspartic acid	C_26_H_37_NO_5_	442.27	3.20	−31.07	Down
neg_4772	Asp Phe Val Glu	C_23_H_32_N_4_O_9_	507.21	3.63	−31.25	Down
neg_21	Solithromycin	C_43_H_65_FN_6_O_10_	889.46	0.47	−31.37	Down
neg_5587	Dioncophylline A	C_24_H_27_NO_3_	799.40	4.89	−31.38	Down
neg_3144	Muzanzagenin	C_27_H_38_O_5_	477.25	2.06	−31.50	Down
neg_3128	Hypochoeroside A	C_21_H_32_O_9_	449.18	2.02	−32.02	Down
neg_3713	Undecylprodigiosin	C_25_H_35_N_3_O	428.25	2.73	−32.17	Down
neg_3530	Cinobufotalin	C_26_H_34_O_7_	493.21	2.52	−32.48	Down
neg_1823	2-(Ethylamino)-4,5-dihydroxybenzamide	C_9_H_12_N_2_O_3_	437.17	1.64	−32.48	Down

#### 3.5.2 Volcanic map of differential metabolites

**Figure 7:**
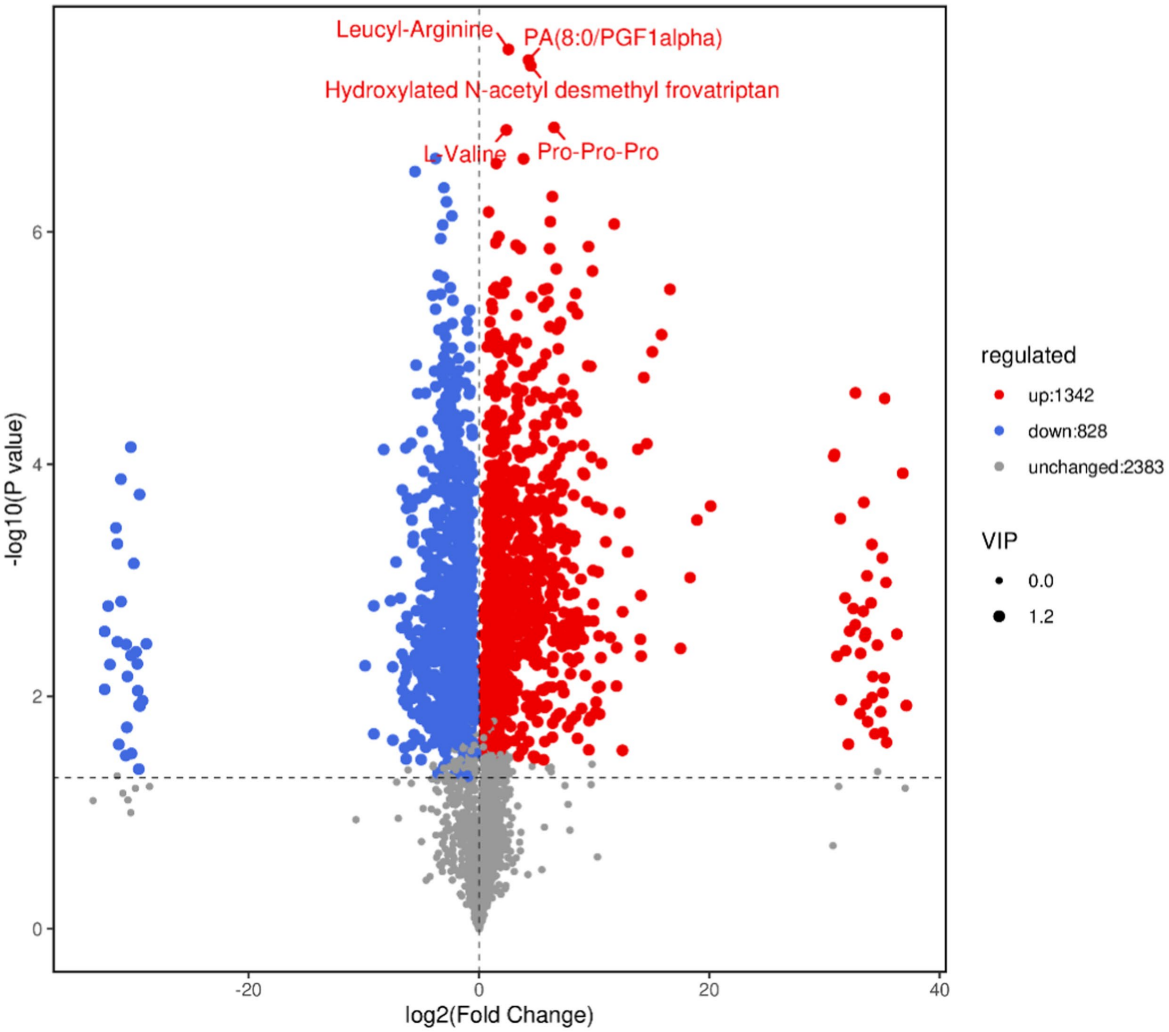
Volcano map of differential metabolites. In the volcano map, each point represents a metabolite. The *x*-axis represents the fold change of different substances in the comparison group (log_2_ transformed), and the *y*-axis represents the *p* value (log_10_ transformed). The size of the scatter point indicates the VIP value of the OPLS-DA model. Larger scatter points correspond to higher VIP values, indicating more reliable selection of differentiated expression metabolites.

The volcano plot can directly show the overall distribution of the difference in metabolite content in the two groups, and the statistical significance of the difference in metabolite content. As shown in Figure 7, blue dots represent downregulated differentially expressed metabolites, red dots represent upregulated differentially expressed metabolites, and gray dots represent metabolites with insignificant differences. After sorting by *p* value, the first five metabolite names identified are shown in the Figure 7.

#### 3.5.3 Differential metabolite cluster analysis

**Figure 8:**
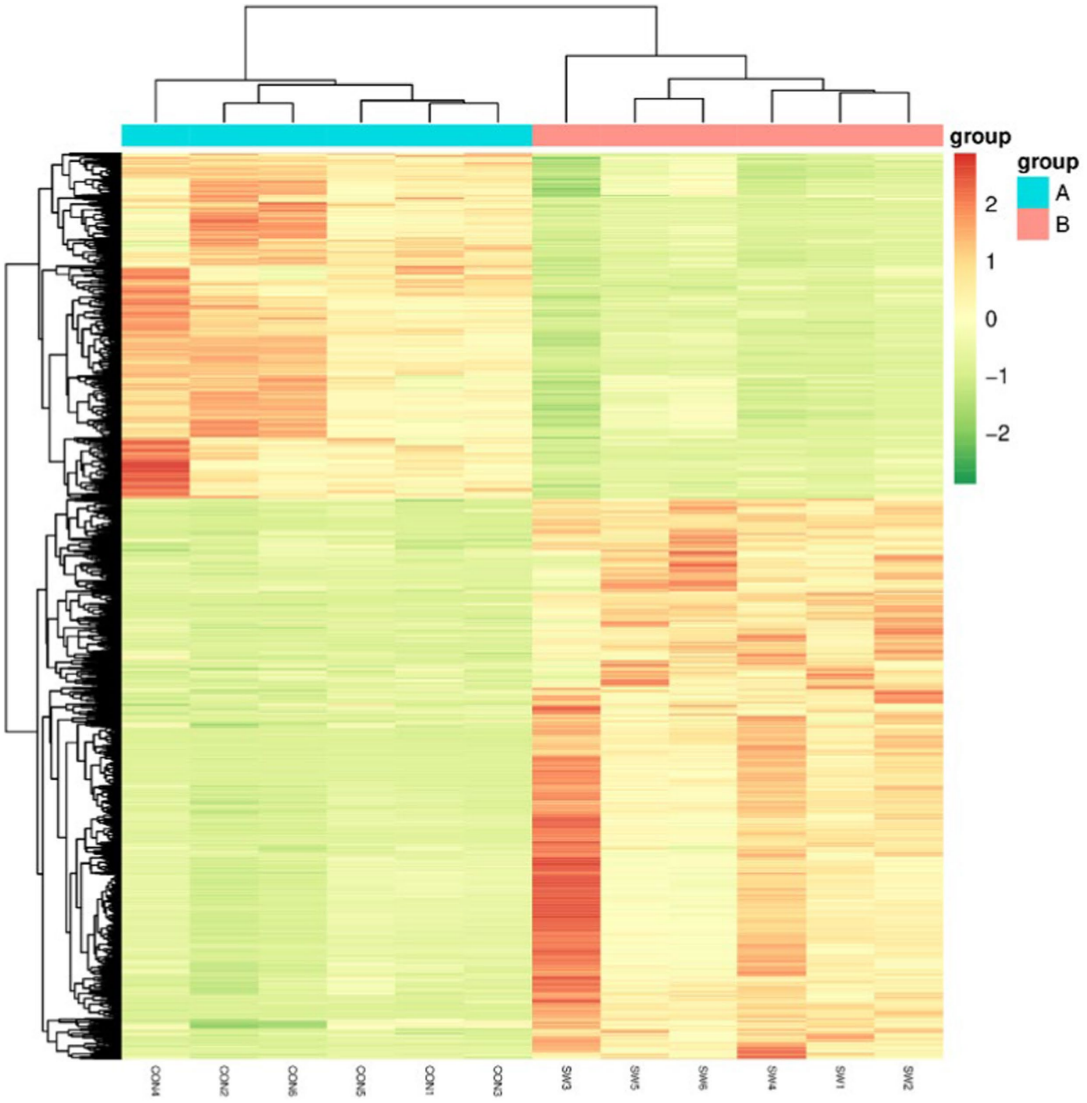
Differential metabolism clustering heat map. Group A served as the control group, while Group B was treated with SW. On the *x*-axis, each sample is represented, and the *y*-axis represents the quantitative value of metabolites standardized by *Z*-score after hierarchical clustering.

Cluster Analysis is a common multivariate statistical analysis method, the quantification of metabolites is expressed by color, the redder the color, the higher its content in a sample, and conversely, the greener the color, the lower the content, which is usually simple and intuitive to observe the overall characteristics of the data. Hierarchical cluster analysis was performed on the differential metabolites, and the results were shown in Figure 8. As can be seen from the figure, the color difference of the selected metabolites in the group is small, indicating that the content is similar, and the color difference between different treatment groups is large and the change is obvious, indicating that these different metabolites can clearly distinguish the control group and the SW group.

### 3.6 Enrichment analysis of differential metabolite metabolic pathways

**Figure 9:**
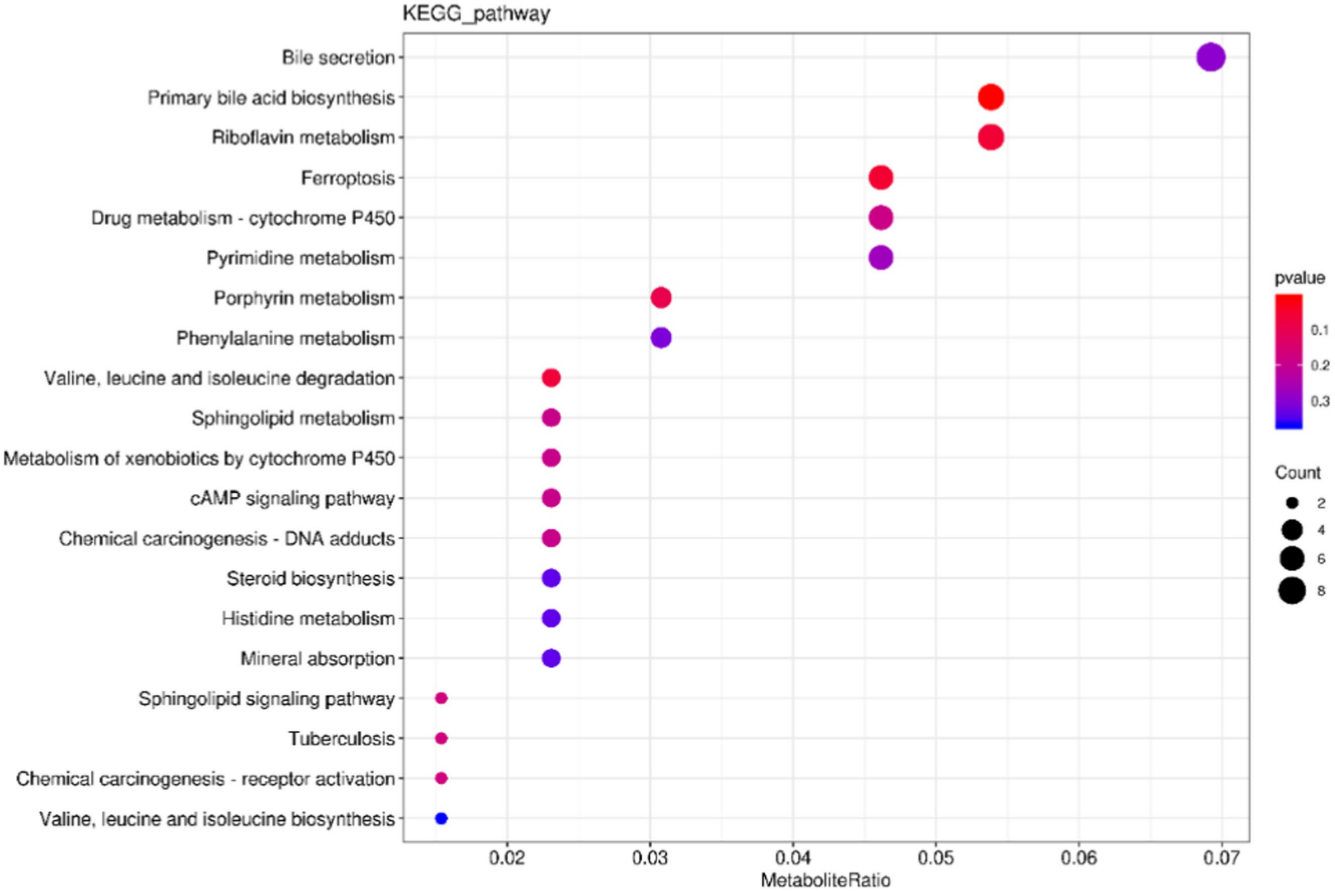
Kyoto Encyclopedia of Genes and Genomes enrichment map of differential metabolites. The *X*-axis represents the ratio of the number of differentially expressed metabolites in the corresponding pathway to the total number of metabolites detected and annotated by the pathway. The *Y*-axis corresponds to the pathway names. The color depth of the dots represents the log *p* value, with a deeper shade of red indicating a more significant enrichment. The size of the dots indicates the number of enriched differentiated metabolites.

The cluster profiler was used to analyze the annotation results of the selected differential metabolites in KEGG by means of hypergeometric test, and the differential metabolite enrichment diagram was drawn (only the top 20 results were displayed). The results were shown in Figure 9, where the size of the points represented the number of enriched differential metabolites. The color of the dots represents *p* value, and the redder the color, the more significant the enrichment. The top 6 channels enriched to Bile secretion, Primary bile acid biosynthesis, Riboflavin metabolism, Ferroptosis, Drug metabolism-cytochrome P450, and pyrimidine metabolism. The five pathways with the most significant differences are Primary bile acid biosynthesis, Ferroptosis, Riboflavin metabolism and Valine, leucine, and isoleucine degradation.

### 3.7 Screening for specific metabolites associated with the SW toxicity

Based on metabolic specificity and the size of VIP value and log-fold change (LFC) of the difference multiple was taken into account. Among them, we have identified five metabolites associated with SW toxicity which were shown in Table 3 and diagnostic accuracy was evaluated by the area under the curve.

**Table 3:** Differential metabolites associated with renal tubular epithelial cells treated by SW.

Number	#ID	Name	VIP	*p*-value	log_2_FC	Regulated	AUC
1	pos_789	Swainsonine	1.4512	0.0000044	5.6368	UP	1
2	pos_17585	3alpha,7alpha-Dihydroxy-5beta-cholestanate	1.4468	0.0000013	3.2384	UP	1
3	pos_1797	2-Hydroxyiminostilbene	1.4176	0.0000458	4.8365	UP	1
4	pos_14725	Glycochenodeoxycholate	1.3034	0.0015693	3.4498	UP	1

## 4 Discussion

This study utilized untargeted metabolomics to analyze the differential metabolites in renal tubular epithelial cells treated with SW compared to the control group. A total of 2,170 significantly different metabolites were screened, most of which were not annotated due to limited related research. The annotated differential metabolites were mainly enriched in the following pathways: Bile secretion, Primary bile acid biosynthesis, Riboflavin metabolism, Ferroptosis, Drug metabolism-cytochrome P450, and Primidine metabolism. Among these differential metabolites, SW is the most distinctive one. However, due to its short half-life of only 20 h (9, 12), it may not be detectable, thus requiring corroboration with other indicators. Therefore, combining VIP values and Log_2_FC, we have screened out substances such as 3alpha,7alpha-Dihydroxy-5beta-cholestanate, 2-Hydroxyiminostilbene, and glycochenodeoxycholate as potential diagnostic values for SW poisoning.

Bile secretion and Primary bile acid biosynthesis were the pathways with the highest number of differentially expressed metabolites. Among them, glycochenodeoxycholate, a component of primary bile acids and a raw material for bile acid synthesis, 3α,7α,12α,26-tetrahydroxy-5β-cholestane, and 7a-Hydroxy-cholestene-3-one, intermediates in the bile acid synthesis pathway (28), were significantly elevated. Previous results genomic study showed consistent enrichment in bile metabolism and drug metabolism-cytochrome P450 when rat renal tubular epithelial cells were exposed to the same conditions as in this study. Additionally, Fu et al. (25) demonstrated that SW induced liver inflammation by altering bile acid metabolism and intestinal microbiota in mice. Thus, these results suggest that exposure to SW may significantly alter the bile acid metabolism whether these changes is associated with the SW-induced cellular damage remain to be investigated.

Cytochrome P450 is one of the most common enzyme families involved in metabolism of the xenobiotics, playing a crucial role in the metabolism of drugs, toxins, and endogenous substrates (29). It plays a crucial role in the clearance of compounds such as drugs, toxins, and endogenous substrates. Our results have found significant increases in 2-Hydroxyiminostilbene, carbamazepine iminoquinone, carbamazepine-O-quinone, morphine-6-glucuronide, and 6-Methylmercaptopurine. These metabolites are associated with the metabolism of Carbamazepine, morphine, and capecitabine, and they rely on P450 enzymes for catalysis (30), suggesting that cells may activate drug metabolism-cytochrome P450 to facilitate the clearance of drugs after exposure to SW. The particular cytochrome P450 induced by the SW however need to be further investigated.

Ferroptosis is a new type of cell death discovered in recent years. Many studies have shown that ferroptosis is related to kidney damage (31–33). A notable elevation in glycochenodeoxycholate (GCDA) levels has been observed in patients suffering from chronic kidney disease. Wang et al. (34) demonstrated that that glycochenodeoxycholate GCDA has the capacity to activate the expression of hepcidin, profoundly influencing the iron homeostasis within the body. Disruption of this iron balance can lead to an excessive accumulation of iron ions, particularly Fe^2+^, which can subsequently trigger ferroptosis (34). Nevertheless, the intricate relationship between GCDA and ferroptosis remains to be thoroughly elucidated and validated.

There are several documented research on related toxicants. For example, another indole alkaloid, vincristine, its impact on plasma metabolites in human children was mainly enriched in the pathways of purine metabolism, arginine biosynthesis, sphingolipid metabolism, glutathione metabolism, glycerophospholipid metabolism, and lysine degradation (35) This differs significantly from the metabolic pathways affected by SW, possibly due to differences in species and tissues of the samples. Rao et al. (36) found that exposure to Matrine in mice resulted in significant changes in metabolites such as cholic acid, taurocholic acid, L-tyrosine, flavin mononucleotide (FMN), UDP-glucuronate, urea, sulfate, and inosine monophosphate, where riboflavin metabolism, purine metabolism, and ascorbate and aldarate metabolism were the most significant pathways. In our results, we also observed significant changes in FMN and UDP, which are involved in riboflavin and pyrimidine metabolism, respectively, suggesting the potential importance of these pathways in the metabolism of alkaloid toxins.

Riboflavin is a component of vitamin B complex and plays an important role in maintaining body health. Flavin mononucleotide (FMN), a coenzyme of riboflavin, is involved in reactions, including drug, lipid and heterogeneous metabolism, energy metabolism, and cellular processes such as protein folding and cell signaling (37, 38). Cytochrome P450 is one of the most common enzyme families involved in drug metabolism, most P450 enzymes use NAD(P)H as a coenzyme, which undergoes dehydrogenation to form NAD(P) and release electrons. The electron is then transferred to FMN, converting it to FMNH+2 (39). These results suggested that SW might induce cell death by inducing ferroptosis, riboflavin metabolism, and drug metabolism-cytochrome P450.

Untargeted metabolomic study can provide the broadest coverage of metabolites and help undiscovered specific metabolites associated with SW toxicity, giving us an objective and comprehensive understanding of the link between the body’s metabolism and external stimuli. It has been shown to be useful in studying the complex diseases. For example, metabolomics has now become a good exploration tool in psychiatry (40, 41). In veterinary medicine, these characteristic metabolic changes related to livestock plant poisoning have great practical application. Clinical symptoms in livestock often have certain specificity but only show when poisoning is severe enough, while these specific changes in the metabolites can help diagnosis of earlier and faster (7), so as to respond more quickly taking corresponding measures to minimize losses. Our study shows that after SW treatment, based on metabolic specificity and the VIP values and Log_2_FC of differential metabolites, we have identified five metabolites which may be used for diagnosis for SW poisoning.

In summary, the findings of this study reveal that the primary metabolites encompass benzenoids, lipids and lipid-like molecules, nucleosides, nucleotides, and analogs, along with organic acids and derivatives. Notably, the differential metabolites are predominantly enriched in specific pathways such as bile secretion, primary bile acid biosynthesis, riboflavin metabolism, ferroptosis, drug metabolism-cytochrome P450, and primidine metabolism. Upon screening for potential biomarkers of SW poisoning, it has been observed that each metabolite exhibits an area under the ROC curve (AUC) value were 1, indicating a significant distinction between the control group and the SW group. Consequently, these metabolites are viable candidates for potential biomarkers of SW poisoning. However, the diagnostic values of these metabolites for SW poisoning need to be further validated in a variety of livestock and to rule out the potential effects of species, feeding environment, grazing practices, and other factors including the coexisting diseases.

Certainly, this study has certain limitations as it was conducted solely as an *in vitro* experiment, unable to fully simulate the *in vivo* environment. In the future, *in vivo* experiments could be conducted on the target animal, sheep, with various time gradients set to more precisely screen for early biomarkers of SW poisoning, thereby improving the detection and prevention of locoweed poisoning.

## Data availability statement

The data presented in the study are deposited in the EMBL-EBI MetaboLights database with the identifier MTBLS9951.

## Ethics statement

The animal study was approved by Laboratory Animal Management and Ethics Committee of Northwest A&F University. The study was conducted in accordance with the local legislation and institutional requirements.

## Author contributions

SZ: Formal analysis, Methodology, Validation, Writing – original draft. YiZ: Methodology, Writing – original draft. HY: Methodology, Writing – original draft. YL: Formal analysis, Writing – original draft. LT: Software, Writing – original draft. YaZ: Software, Writing – original draft. PS: Formal analysis, Writing – original draft. KW: Software, Writing – original draft. BZ: Conceptualization, Writing – review & editing. HL: Conceptualization, Funding acquisition, Project administration, Resources, Supervision, Visualization, Writing – review & editing.

## Footnotes

^1^ https://hmdb.ca/

^2^ http://www.genome.jp/kegg/
